# The Usefulness of the Application of Compression Therapy among Lipedema Patients-Pilot Study

**DOI:** 10.3390/ijerph20020914

**Published:** 2023-01-04

**Authors:** Monika Czerwińska, Jacek Teodorczyk, Dawid Spychała, Rita Hansdorfer-Korzon

**Affiliations:** 1 Department of Physiotherapy, Medical University of Gdańsk, 80-211 Gdańsk, Poland; 2 Department of Nuclear Medicine and Radiology Informatics, Medical University of Gdańsk, 17 Mariana Smoluchowskiego Street, 80-214 Gdańsk, Poland

**Keywords:** lipedema, compression therapy, subcutaneous adipose tissue, ultrasonography, quality of life, circumference, complete decongestive therapy

## Abstract

(1) Background: Although lipedema has gained more interest among researchers, specific treatment methods are still unknown. This study aims to identify the effects of compression therapy combined with exercises compared to exercising only. Moreover, the aim is to assess the methodology and outcome measurements before conducting a larger study. (2) Methods: Six women with lipedema were enrolled in the study; three were undergoing exercise program and compression therapy using compression leggings, and the remaining three were undergoing exercises only. During the first 4 weeks, intervention was under the supervision of a physiotherapist, and in the remaining weeks, participants were exercising independently. Measurements of circumference, weight, thickness of the skin and adipose tissue, symptom severity, and quality of life were taken at baseline, after 4 weeks and after 6 weeks; (3) Results: There was a significant decrease in the subjectively reported tendency for bruising and pain at palpation among patients that received compression therapy. Additionally, there was a tendency to reduce or maintain the circumference of the legs in patients using compression, while it tended to increase in patients without compression. (4) Conclusions: Preliminary results indicate that compression therapy, combined with exercises, could improve the quality of life and decrease the severity of lipedema symptoms. Further studies on a large clinical group are advisable.

## 1. Introduction

Lipedema is a chronic disease of adipose tissue causing excessive accumulation of fatty tissue around the lower and/or upper extremities [1]. It leads to a visible disproportion between slim trunk and enlarged limbs. The characteristic feature of lipedema is that the adipose tissue is symmetrically accumulated around both extremities and that distal parts of the limbs are not affected. It leads to the occurrence of characteristic folds above the ankles [2,3]. Although lipedema is gaining more and more interest among researchers, there are still some misconceptions regarding this condition [4,5]. It is currently stated that a combination of genetic factors, hormonal changes, and microvascular disorders are responsible for lipedema occurrence; however, an unequivocal cause has not yet been established [6]. Moreover onset of lipedema symptoms could be triggered by traumatic events or other psychological distress [7,8]. Lipedema almost exclusively affects women, and its onset oscillates around puberty, pregnancy, or menopause [2,9]. Some researchers present an estimated prevalence of lipedema of 1:72,000, but since lipedema is not fully known, the specific incidence cannot be indicated [10].

Currently, clear diagnostic methods or protocols for lipedema are unavailable, so the diagnosis is made upon physical examination [11,12]. Misunderstanding and lack of knowledge of this condition often cause incorrect diagnosis. In many cases, the correct diagnosis is made after many years of the onset of lipedema, when the patient has significant limitations [13,14]. Lack of proper education and early conservative treatment often causes severe lipedema, which leads to disability.

Lipedema is not only an aesthetic issue. Patients affected by this condition experience various ailments that have an immense impact on their daily functioning. Apart from disproportionate body shape, women with lipedema suffer from heaviness in the affected areas and pain at palpation. Pain and tenderness of lipedema tissue are thought to be caused by inflammation in subcutaneous tissue. The presence of inflammation in lipedema could be indicated by increased sodium content in the skin and proinflammatory macrophages that produce cytokines (interleukin-1β (IL-1β), IL-6, IL-12, IL-23, and TNF-α) in adipose tissue; however this data has not been fully confirmed yet [15,16]. Although easy bruising is widely reported in lipedema, the exact reason for its existence among lipedema patients has not yet been established [17]. Moreover, lipedema patients also struggle with a lack of understanding, low self-esteem, feeling of hopelessness, and a decreased level of quality of life [18,19,20,21].

Formerly described treatment methods were based on lymphedema treatment, i.e., Complete Decongestive Therapy (CDT), which consists of Manual Lymphatic Drainage (MLD), compression therapy, movement therapy, and skincare [22,23]. However, the traditional anti-edematous treatment of lipedema is currently being questioned, because in pure lipedema there is no actual fluid accumulation [4,24]. Certainly, in the case of lipo-lymphedema, the use of CDT is indicated, but in the case of pure lipedema, only some elements of CDT are applicable. Currently, compression therapy is considered to be a suitable treatment method due to its anti-inflammatory effect, but more research is needed to fully confirm this statement [4,24]. Compression therapy does not directly reduce adipose tissue; however, it is thought to be useful in alleviating symptoms. A recent study shows that the longer patients wear compression garments during the day, the higher the reduction of symptoms [25].

The selection of appropriate tools is required to evaluate the therapeutic effectiveness of new methods in the treatment of lipedema. The currently used methods are measurements of circumference, volume, body weight, BMI, WHR, ultrasonography, and subjective scales regarding the severity of symptoms and quality of life. Exploring the most accurate tool will enable the subjective assessment of the effect of treatment and compare the results between different institutions, which is vital to choosing the most effective treatment method [26].

The study aims to compare the use of two methods of lipedema treatment: physical exercises combined with compression therapy and physical exercises only. In addition, this study aims to identify potential tools that would help objectively assess the effectiveness of non-surgical therapeutic methods. The pilot study is primarily aimed at checking the correctness of the selection of measurement methods and evaluating the methodology of the study before conducting larger-scale research.

## 2. Materials and Methods

### 2.1. Patients

The participants were recruited from social media groups for women and an outpatient physiotherapeutic clinic. All participants had to give written consent before the beginning of the study. The ethical consent was obtained from the Medical University of Gdansk Ethical Committee (Approval number-912/2021-2022). Primary qualification consisted of detailed questions asked to participants regarding inclusion criteria: female sex, age between 18 and 60 years old, no contraindications to physical activity and compression therapy, and reporting the presence of at least one of the following symptoms (disproportion between the trunk and lower limbs, heaviness in legs, pain at palpation, easy bruising, difficulty losing weight). The exclusion criteria for this study were: male sex, pregnancy, orthopedic trauma in the last 2 years’ time, cancerous disease in the last 5 years’ time, venous thrombosis, exacerbation of chronic diseases, contraindications for exercises, and compression therapy. Secondary qualification was based on a physical examination, which assessed lipedema-specific symptoms. The examination consisted of a visual assessment of body proportion, fat tissue distribution, skin condition, bruising and the palpation of tissue assessing sensitivity to touch and consistency. Additionally, tests differentiating lipedema from other diseases were conducted (Stemmer sign, pitting test, and temperature). Patients were also asked to answer questions regarding diet, lifestyle, physical activity, and the onset of their symptoms.

### 2.2. Intervention

**Figure 1:**
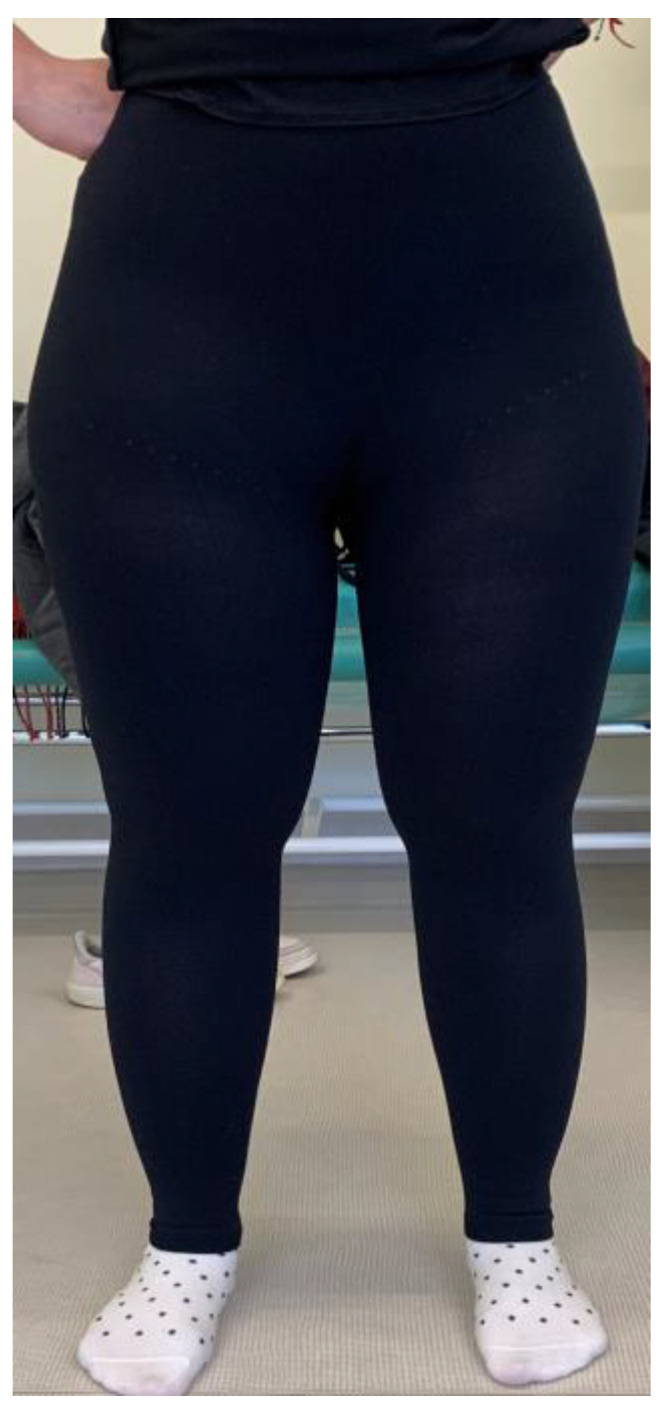
Lipedema patient wearing compression leggings.

All participants were randomly divided into two groups. Group A was undergoing an exercise program, and group B was undergoing an exercise program combined with compression therapy. Three participants received flat-knitted class 2 compression leggings. Due to the use of an innovative, seamless and breathable knitted fabric, these leggings are characterized by high comfort. The three-dimensional, soft fabric perfectly adapts to anatomical traits, allowing an optimal degree of compression. (Figure 1)

The entire study lasted 6 weeks. In the first 4 weeks, participants underwent an exercise program 3 times a week—1 × week under the supervision of a physiotherapist, and 2 × solely at home. Group B was advised to wear compression leggings at least 12 h per day, especially during activity. In the remaining 2 weeks, patients underwent an exercise program and using compression garments independently without supervision. Patients were advised not to change their dietary habits and not to attend any other physical therapy during the study. The exercise protocol is available on request from the corresponding author.

### 2.3. Evaluation

The evaluation of effects consisted of circumference measurement, body weight measurement, subjective symptom severity questionnaire, skin and adipose tissue thickness measurement using ultrasonography, and quality of life assessment using the SF-36 questionnaire. The evaluation was done before the study, after 4 weeks, and after 6 weeks. Body circumference was collected at certain reference points as follows:

P5—waist circumference in the narrowest point

P7—hips in the widest point

P8—thigh at 2/3 length from knee to the buttock

P9—thigh at 1/3 length from knee to the buttock

P10—mid patellar circumference

P11—calf at 2/3 length from the ground to the knee

P12—calf at 1/3 length from the ground to the knee

P13—foot circumference at the level of the metatarsal heads

Waist and hip measurements were used to calculate WHR, and body weight was measured to calculate BMI.

The thickness of skin and adipose tissue was measured using ultrasonography (Honda Electronics HS-2100, Toyohashi, Japan) at four certain reference points: ½ length in the front of the thigh; ½ length in the front of the shin; ½ length on the side of the shin; above the medial malleolus.

To minimize the risk of excessive pressure of the ultrasonic probe against the tissue, which could distort the results, a large amount of gel was used for the test.

In the symptom-severity survey, patients were subjectively rating how on a scale from 0 to 10 certain lipedema ailments impact their life. They were rating symptoms such as the level of experiencing pain, disproportion between slim trunk and enlarged limbs, swelling of the legs, heaviness in legs, tendency to bruising, pain at palpation, fat accumulation mostly around lower limbs, and daily activities restrictions.

The SF-36 questionnaire was used to assess the quality of life of patients in eight dimensions: physical functioning, role limitations due to physical health, role limitations due to emotional problems, energy/fatigue, emotional well-being, social functioning, pain, and general health. The scale ranges from 0 (worst) to 100 (best) in each of the above mentioned dimensions.

### 2.4. Statistical Analysis

Statistical analyses were carried out using the SPSS Statistics for Windows, version 26 (IBM Corp., Armonk, NY, USA) to perform a two-factor analysis of variance in a 3 × 2 mixed scheme. Due to the very small sample size, the significance level in this study was α = 0.10. To test the hypothesis regarding the effect of the type of therapy used on selected indicators of lipedema, a series of two-way analyses of variance (ANOVA) was performed in a mixed scheme 3 (measurement 1 vs. measurement 2 vs. measurement 3) × 2 (group A vs. group B), wherein the first of these factors is within-subjects, and the second is between-subjects. The dependent variables were the following measurements: BMI, subjective assessment of the severity of lipedema symptoms, WHR, measurements of body circumferences at specific points, measurements of skin and subcutaneous tissue thickness using ultrasound, and assessment of the quality of life using the SF-36 form.

## 3. Results

### 3.1. Participants

**Figure 2:**
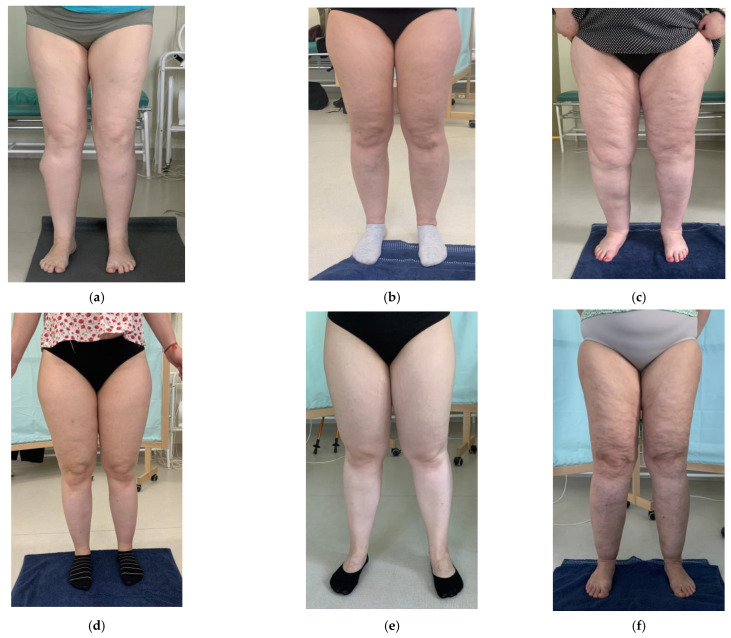
Lipedema patients from group A (**a**–**c**) and from group B (**d**–**f**).

Six women with lipedema were included in this study. All of the participants were randomly divided into 2 groups—group A (*n* = 3) received only exercises and group B received exercises combined with compression therapy. All participants from group B claimed to wear compression leggings every day as suggested during the first 4 weeks; however during the remaining 2 weeks patients failed to use compression every day. The mean time of wearing compression reported by patients was approximately 11 out of 14 days. Patients from group A and B are presented on the Figure 2. The characteristics of the participants in presented in Table 1.

### 3.2. Circumfernce, BMI, WHR, Body Weight

Body circumference was measured in eight reference points. Table 2 presents the results of the circumference measurement of each patient.

Changes in circumference were not significant; however, even a slight reduction or maintaining the same value is perceived as beneficial. The results show that, in group A the total growth of circumference of the thigh at 2/3 length from knee to buttock (P8) comparing 6 weeks to baseline, was 4 cm on one side, while in group B, a total decrease of 1 cm could be observed. The measurement P11 (calf at 2/3 length from the ground to the knee) in the group that underwent compression therapy did not change during the whole study, while in the group without compression, there was an increase of 0.5 cm on each side in patient 1, and patient 2 experienced an increase by 1 cm on the right side, and patient 3 had +1 cm on the left side after 6 weeks compared to baseline. The circumference of the calf at 1/3 length from the ground to the knee (P12) was reduced by 2 cm on each side in patient 3 from the group with compression after 4 weeks; however, after 6 weeks, it increased by 1 cm on the right and 0.5 cm on the left side. The remaining two patients from group B did not experience significant change comparing circumference at the beginning to the end of the study. In group A, measurement P12 did not change in patient 1; patient 2 experienced an increase of 2 cm on each side, and patient 3 had a decrease of 1 cm on the right and an increase of 1 cm on the left side. Circumference P13 (foot circumference at the level of the metatarsal heads) did not change in any of the patients, which confirms the statement that feet are not affected by lipedema.

It should be noted that one of the patients from group B (patient 1B) reported that she was menstruating during the second measurement. An increase of approximately 1 cm could be observed among this patient in P10 and P12 measurements on both limbs after 4 weeks, while neither of the remaining participants had considerable growth in this parameter. After 6 weeks, the above-mentioned parameter returned to baseline level in patient 1B. Additionally, patient 1B had a body weight increase of 0.8 kg after 4 weeks, which declined at 6 weeks (−1.3 kg).

Circumferences P5 (waist) and P7 (hips) were used to calculate waist-to-hip ratio. This ratio is an indicator of body shape and is useful to evaluate the changes in the disproportion between the lower and upper body in lipedema patients. An increase in WHR stands for decreasing disproportion, and a reduction in WHR suggests increasing disproportion. The general tendency towards the reduction of WHR was observed in group A (0.78 at baseline, 0.77 after 4 weeks, and 0.76 after 6 weeks), while in group B there was a tendency to increase in this ratio (0.74 at baseline, 0.74 after 4 weeks, and 0.75 after 6 weeks. This can suggest that the disproportion between trunk and hips declined among patients that underwent an exercise program combined with compression, while it deepened among patients that underwent only an exercise program.

Body weight increased among all of the patients by approximately 1 kg, both in groups A and B. However it should be noted that, despite the rise in weight, there was a tendency to maintain or reduce circumference in lower limbs among patients from group B, while in group A, there was a general tendency to increase the circumference in lower extremities.

### 3.3. Symptom Severity

The subjective level of experiencing lipedema symptoms was assessed before the intervention, after 4 weeks, and after 6 weeks. Participants were asked to rate how severely they experience certain lipedema features on a scale from 0 to 10, where 0 was no symptom and 10 was the highest possible intensity of the symptom. Additionally, patients were asked to rate the general level of pain on a VAS scale (0–10) and how the symptoms affect their daily functioning (0–10). The results are presented in Table 3.

Statistically significant differences between groups A and B were observed within three features: tendency to bruising, pain at palpation, and the impact of lipedema symptoms on daily functioning. In the group that underwent an exercise program combined with compression (group B), there was a reduction in the subjective level of a tendency to bruising after 4 weeks from 5.38 to 1.67, and after 6 weeks, the intensity of this feature was rated at 1.33, whereas, in the group that underwent only exercise program, the level of experiencing bruising decreased slightly from 5.67 to 5 after 4 weeks; however, after 6 weeks, the intensity of this feature increased to 7/10.

The reduction in the intensity of pain at palpation was 27% after 4 weeks and 64.2% after 6 weeks from baseline in a group with compression, while in the group without compression, there was an increase in the intensity of pain at palpation of 9% after 4 weeks and 63% after 6 weeks from baseline. The impact of lipedema symptoms on daily functioning decreased in group B from 1.67 to 0.67 after 4 weeks and increased back to 2 after 6 weeks; however, in group A after 4 weeks, it increased from 5 to 6 and decreased to 4.67 after 6 weeks.

Although changes in other symptoms were not statistically significant, some tendencies could be observed. The severity of swelling around the ankles and heaviness in lower extremities was higher after 4 weeks in the group without compression (11.1% and 10.4%, respectively), while in a group with compression therapy, there was a reduction of 30.2% and 34.1% in the intensity of those symptoms. After 6 weeks, a slight decrease was observed in swelling and heaviness in legs among participants from group A (4.76% and 14%), and patients from group B still reported a reduction of symptoms (11% and 18.25% in swelling and heaviness in legs, respectively).

### 3.4. Quality of Life

Quality of life was assessed before the intervention, after 4 weeks, and after 6 weeks using the SF-36 questionnaire. It is a 36-item survey evaluating the quality of life in eight different dimensions: physical functioning, role limitations due to physical health, role limitations due to emotional problems, energy/fatigue, emotional well-being, social functioning, pain, and general health. The results of quality of life among participants are presented in Table 4. The score for each dimension ranges from 0 (the worst possible quality of life) to 100 (the best possible quality of life).

**Figure 3:**
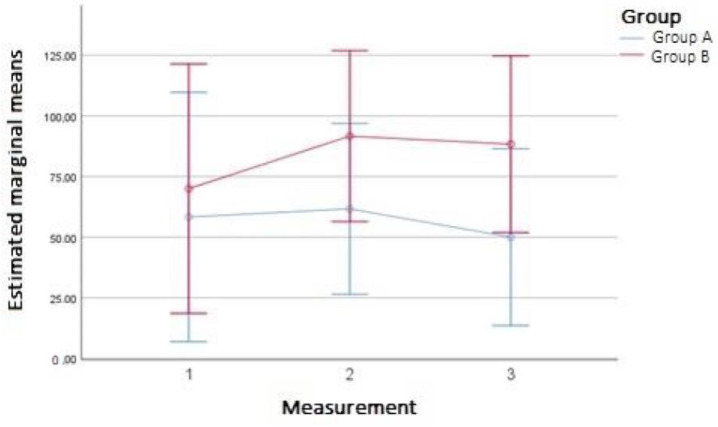
Graph presenting changes in SF-physical functioning in groups A and B over time: measurement 1 (baseline), measurement 2 (after 4 weeks), measurement 3 (after 6 weeks).

Regarding Sf-36’s measure of physical functioning, the intragroup effect regarding the moment of measurement (regardless of the group) turned out to be statistically significant, and post-hoc comparisons showed that physical functioning was higher in measurement 2 (after 4 weeks) compared to measurement 3 (after 6 weeks) (*p* = 0.047). Moreover, the increase after 4 weeks was higher (31%) in the group with compression therapy compared to the group that underwent exercises only (5.7%) (Figure 3).

Sf-36’s measure of the energy/fatigue intra-group effect (regardless of the group) turned out to be statistically significant, and post-hoc comparisons showed that the score was higher in measurement 2 compared to measurement 1 (*p* = 0.055) and measurement 3 (*p* = 0.015). The higher score, the higher level of energy and the lower level of fatigue. This shows that the level of energy was the highest after 4 weeks (82.5 in group A and 70.3 in group B).

Although in total, the SF-36 results were not statistically relevant, some trends could be observed. In group A, there was no change in the total score after 4 weeks, and after 6 weeks, quality of life decreased by 2.4%, while in group B, there was an increase by 6% after 4 weeks, and then a decrease by 2.6% after 6 weeks. Despite the reduction in quality of life between the second (4 weeks) and third (6 weeks) measurement, there was an increase of 3.2% from baseline until the end of the study.

### 3.5. Ultrasonography

The thickness of the skin and adipose tissue was measured using ultrasonography three times: before intervention (measurement 1), after 4 weeks (measurement 2), and after 6 weeks (measurement 3). To eliminate the risk of error, the measurements were taken at certain reference points: ½ length in the front of the thigh, ½ length in the front of the shin, ½ length on the side of the shin, and above the medial malleolus; those points were carefully marked before every measurement. The thickness of the skin did not vary significantly between groups and did not change during the therapeutic process; mean values of the skin thickness were as follows: 1.85 mm, 1.6 mm, 1.6 mm, and 1.59 mm for thigh, front shin, side shin, and above medial malleolus, respectively. In group A, the mean increase in the thickness of the adipose tissue in the front of the thigh was 1.4 mm comparing baseline to the end of the study, while in group B, it remained at the same level.

The mean thickness of adipose tissue in the front of the shin in group A at baseline was 28.55 mm. It increased by 1.335 mm after 4 weeks and then decreased by 1 mm, whereas in group B, there was no difference in this measurement after 4 weeks, and after 6 weeks, there was a decrease of 0.7 mm. Mean thickness at baseline in group B was 27 mm.

The thickness of adipose tissue on the side of the shin increased slightly in both groups, 0.365 mm in group A and 0.435 mm in group B after 4 weeks.

**Figure 4:**
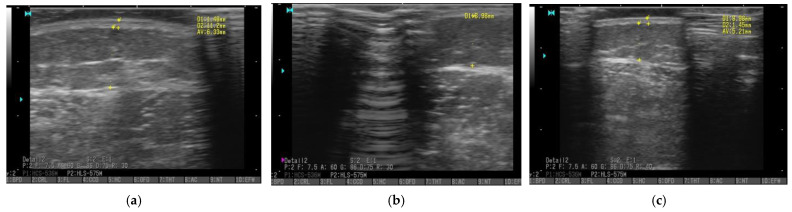
Thickness of adipose tissue measured by ultrasonography at ½ length on the side of the shin on the right leg in Patient 2B at baseline (**a**), after 4 weeks (**b**), after 6 weeks (**c**). The thickness of adipose tissue is marked with † signs, ^1^ and ^2^ measurement stand for the thickness of the skin.

The last parameter, thickness of adipose tissue above the ankle, in group A did not change significantly and was 19.95 mm, 19.065 mm, 19.75 mm during the 3 measurements, respectively. In group B, it declined after 4 weeks from 18.3 mm to 17.26 mm and increased to 18.2 mm after 6 weeks. Although the changes in the thickness of adipose tissue were not statistically significant (>0.1), even a small reduction or a lack of growth of adipose tissue thickness is a valuable effect. Figure 4 presents the thickness of adipose tissue on the side of the shin measures at baseline, after 4 weeks, and after 6 weeks.

## 4. Discussion

Lipedema is undoubtedly a multifactorial and complex condition that not only causes disproportionate body shape but also leads to painful ailments [14,27]. Even though the awareness of this disease has been rising in the past few years, it is still an unrecognized problem [6]. None of the participants had previously been diagnosed with lipedema, while all of them experienced characteristic symptoms.

When it comes to conservative treatment, the currently suggested therapeutic approach focuses mostly on the reduction of the symptoms and preventing the progression of the disease [7,11]. Some researchers had previously examined the effectiveness of complete decongestive therapy; however, there are no studies assessing the efficacy of compression therapy separately from manual lymphatic drainage. As for the latest reports, manual lymphatic drainage may not be necessary for all lipedema patients, since in pure lipedema, there is no fluid accumulation [4,24]. The mechanical impact of compression therapy on adipose tissue has not yet been established, and more specific research regarding the mechanobiology of adipose tissue is needed [28].

The results of our study show that compression therapy combined with exercises can be beneficial in improving the quality of life and reducing the level of experiencing symptoms.

A survey study by Paling I. and Macintyre L. assessed experiences with compression therapy among lipedema patients. Out of 279 respondents, 54% declared that wearing compression makes a difference in alleviating lipedema symptoms. The same study discovered the correlation between compression-wearing time and its impact on decreasing the severity of lipedema symptoms. Compression was reported to be useful in 68.3% and 66.67% of respondents who wore compression garments every day and 5–6 days a week respectively. However, only 36.67% of people who reported wearing compression 3 to 4 days a week found it helpful in reducing lipedema symptoms. In our study, during the first 4 weeks, participants wore compression every day; however, during the last 2 weeks, while they had no supervision, they reported not managing to wear leggings every day. This could be the reason for the aggravation of some results (SF-36 Physical Functioning, subjective impact of lipedema on daily activities) between the second and third measurements [25].

Another survey study by Lipoedema UK showed that 52% of lipedema patients wearing compression reported that compression has an impact on pain relief and 76% states that it helps in reducing the size of the affected areas [29].

A study by Atan examined the effectiveness of CDT or IPC or exercises only among 31 lipedema patients. In this study, the SF-36 questionnaire was used to assess the quality of life. The result showed the greatest increase in SF-36’s measure of physical functioning among participants that underwent CDT [30]. Our study also showed significant improvement in SF-physical functioning among all of the participants regardless of treatment method; however, the enhancement was higher in the group that underwent exercises combined with compression.

Ultrasonography has been previously used to monitor the effects of lipedema treatment. The changes in adipose tissue were assessed before and after therapy, and the results revealed a reduction in fibrosis and a decrease in the number of hyperechoic masses in subcutaneous adipose tissue after the intervention [31,32]. Evaluation of the thickness of adipose tissue was also used to support lipedema diagnosis [33]. A study by T. Hirsch showed that the thickness of cutis measured by ultrasonography varied between all participants (*n* = 147); however, those differences were not associated with their condition (lipedema, lymphedema, lipohypertrophy, obesity and healthy women) [34].

As our study is a preliminary pilot study on a small group of patients (*n* = 6), it has some limitations. First of all, a larger-scale study should be conducted to eliminate the possible error due to individual differences. Secondly, even though participants were asked not to change their diet during the study, some patients admitted increasing their caloric intake, so patients should monitor their diet more carefully, as changing habits could have an impact on the results. The phase of the menstrual cycle should also be noted in future research, as it can impact the circumference and weight measurement.

## 5. Conclusions

Lipedema is still an unrecognized, underdiagnosed problem. Early diagnosis and conservative therapy are crucial to prevent serious complications. Women with lipedema experience painful symptoms; 100% of participants report heaviness in the lower limbs, and 84% have pain at palpation.

Assessing quality of life and severity of lipedema symptoms are feasible tools to evaluate the effectiveness of compression therapy combined with physical exercises to exercises only. Our study revealed a greater reduction of symptoms and a higher improvement in quality of life among patients who underwent compression combined with exercises. Therapy without supervision gives a weaker effect than under the control of physiotherapist. There was the aggravation in some parameters of quality of life between the 4-week and the 6-week points, while patients were undergoing therapy solely at home. Our study showed that the circumference of lower extremities tends to increase without compression, while using compression garments helps to maintain and reduce the circumference; however, larger-scale studies are necessary to confirm this statement. The ultrasonography of adipose tissue should be considered in future research as a tool to evaluate treatment effectiveness, since the thickness of the skin did not change among patients, and this measurement could be omitted in future research.

## Figures and Tables

**Table 1:** Characteristics of participants in groups A and B.

Feature	Total (*n* = 6)	Group A (*n* = 3)	Group B (*n* = 3)
Age, mean (SD)	39.7 (12.8)	48 (4.9)	31 (12.8)
BMI, mean (SD)	31.35 (8.4)	34.56 (10.6)	28.12 (2.83)
Body weight (kg), mean (SD)	90.89 (28.64)	104 (34.215)	77.76 (11.19)
Previous lipedema diagnosis			
Yes	0	0	0
No	6 (100%)	3 (100%)	3 (100%)
Lipedema symptoms			
Heaviness in lower extremities	6 (100%)	3 (100%)	3 (100%)
Pain at palpation	5 (83.34%)	3 (100%)	2 (66.67%)
Disproportion between slimmer trunk and enlarged lower limbs	5 (83.34%)	2 (66.67%)	3 (100%)
Easy bruising	5 (83.34%)	3 (100%)	2 (66.67%)
Accumulation of fat tissue mostly around legs	5 (83.34%)	2 (66.67%)	3 (100%)
Characteristic fat cuffs above the ankles	4 (66.67%)	2 (66.67%)	2 (66.67%)
Swelling around the ankles	3 (50%)	2(66.67%)	1 (33.34%)
Lipedema Stage			
Stage 1	2 (33.34%)	1 (33.34%)	1 (33.34%)
Stage 2	3 (50%)	1 (33.34%)	2 (66.67%)
Stage 3	1 (16.67%)	1 (33.34%)	0
Previous dieting			
Yes	4 (66.67%)	2 (66.67%)	2 (66.67%)
No	2 (33.34%)	1 (33.34%)	1 (33.34%)
Declared level of physical activity			
None	1 (16.67%)	1 (33.34%)	0
Low	1 (16.67%)	1 (33.34%)	0
Medium	4 (66.67%)	1 (33.34%)	3 (100%)
High	0	0	0

**Table 2:** BMI, WHR, weight (kg), and circumference (cm) among all patients pre- intervention, after 4 weeks, and after 6 weeks.

Feature	Patient 1A	Patient 2A	Patient 3A	Patient 1B	Patient 2B	Patient 3B
Weight (kg) pre-intervention	114	58	140	69.7	70	93.6
Weight (kg) after 4 weeks	117.5	58	140	70.5	74	93.6
Weight (kg) after 6 weeks	116.8	58.2	140.4	69.2	73.1	93.8
BMI pre-intervention	32.6	22.65	48.44	24.69	28.04	31.63
BMI after 4 weeks	33.6	22.65	48.44	24.9	29.64	31.63
BMI after 6 weeks	33.4	22.73	48.58	24.5	29.28	31.7
WHR pre-intervention	0.84	0.77	0.74	0.64	0.76	0.82
WHR after 4 weeks	0.82	0.75	0.74	0.64	0.75	0.83
WHR after 6 weeks	0.8	0.73	0.75	0.66	0.77	0.81
P5 (cm) pre intervention	99	72	111	66	79	92
P5 (cm) after 4 weeks	98	71	111	64	78	93
P5 (cm) after 6 weeks	97	71	112	66	80	92
P7 (cm) pre intervention	118	93	150	103	104	111
P7 (cm) after 4 weeks	119	94	150	100	104	112
P7 (cm) after 4 weeks	120	96	150	101	104	113
P8 (cm) pre-intervention	R-65	R-54	R-81	R-63	R-63	R-67
	L-65	L-54	L-80	L-63	L-63	L-67
P8 (cm) after 4 weeks	R-66	R-54	R-84	R-64	R-63	R-66
	L-66	L-54	L-83	L-64	L-63	L-66
P8 (cm) after 6 weeks	R-66	R-54	R-84	R-64	R-63	R-65
	L-66	L-54	L-83	L-64	L-63	L-65
P9 (cm) pre-intervention	R-54.5	R-47	R-74	R-52	R-53	R-56
	L- 54.5	L-47.5	L-73	L-52	L-53	L-56
P9 (cm) after 4 weeks	R-55.5	R-47	R-75	R-52.5	R-53	R-56
	L-55.5	L-46.5	L-74	L-53	L-53	L-56
P9 (cm) after 6 weeks	R-56	R-46.5	R-75	R-53	R-53	R-56
	L-56	L-46.5	L-74	L-53	L-53	L-56
P10 (cm) pre-intervention	R-45	R-36	R-58	R-37	R-38	R-45
	L-45	L-36	L-58	L-37	L-38	L-45
P10 (cm) after 4 weeks	R-45	R-36	R-59	R-38	R-38	R-45
	L-45	L-36	L-58	L-38	L-38	L-45
P10 (cm) after 6 weeks	R-45	R-36.5	R-59	R-37	R-38	R-45
	L-45	L-36.5	L-58	L-37	L-38	L-45
P11 (cm) pre-intervention	R-45.5	R-38	R-54	R-42	R-42	R-48
	L-45.5	L-39	L-54	L-42	L-41	L-48
P11 (cm) after 4 weeks	R-45	R-39	R-54	R-42	R-41	R-48
	L-44.5	L-39	L-54	L-42	L-41	L-48
P11 (cm) after 6 weeks	R-46	R-39	R-54	R-42	R-41	R-48
	L-46	L-39	L-55	L-42	L-41	L-48
P12 (cm) pre-intervention	R-30	R-28	R-39	R-28	R-28	R-36
	L-30	L-28	L-37	L-28	L-28	L-36
P12 (cm) after 4 weeks	R-30	R-28	R-39	R-28.5	R-28.5	R-34
	L-30	L-28	L-37	L-29	L-28	L-34
P12 (cm) after 6 weeks	R-30	R-30	R-38	R-27.5	R-28	R-35
	L-30	L-30	L-38	L-28	L-28	L-34.5
P13 (cm) pre-intervention	R-26	R-21	R-26	R-23	R-22	R-24
	L-26	L-21	L-26	L-23	L-22	L-24
P13 (cm) after 4 weeks	R-26	R-21	R-26	R-23	R-22	R-24
	L-26	L-21	L-26	L-23	L-22	L-24
P13 (cm) after 6 weeks	R-26	R-21	R-25.5	R-23	R-22	R-24
	L-26	L-21	L-25.5	L-23	L-22	L-24

**Table 3:** Symptom severity pre intervention, after 4 weeks, and after 6 weeks.

Outcome Measurement	Measurement Time	Group A	Group B	p^1^	p^2^	p^3^
Pain-VAS (0–10)	Pre-intervention	5.67	2.67	0.35	0.22	0.68
	After 4 weeks	5.33	3			
	After 6 weeks	5	2.3			
Subjective impact on daily functioning (0–10)	Pre-intervention	5	1.67	1.0	**0.078**	**0.058**
	After 4 weeks	6	0.67			
	After 6 weeks	4.67	2			
Subjective disproportion between lower and upper body (0–10)	Pre-intervention	6	5.34	1.0	0.68	0.89
	After 4 weeks	6	5.34			
	After 6 weeks	6.34	5			
Subjective swelling around the ankles (0–10)	Pre-intervention	5.67	4.3	0.726	0.412	0.41
	After 4 weeks	6.3	3			
	After 6 weeks	6	2.67			
Subjective heaviness in lower extremities (0–10)	Pre-intervention	6.34	5.67	0.21	0.39	0.23
	After 4 weeks	7	3.67			
	After 6 weeks	6	3			
Subjective tendency to bruising (0–10)	Pre-intervention	5.67	5.33	**0.079**	**0.088**	**0.034**
	After 4 weeks	5	1.67			
	After 6 weeks	7	1.33			
Subjective pain at palpation (0–10)	Pre-intervention	3.67	4.67	0.78	0.69	**0.016**
	After 4 weeks	4	3.33			
	After 6 weeks	6	1.67			
Subjective level of accumulation of fat tissue mostly around legs (0–10)	Pre-intervention	7.67	7	0.43	0.46	0.64
	After 4 weeks	7.33	5.3			
	After 6 weeks	7.33	5			

p^1^—effect of measurement (pre-intervention, after 4 weeks, after 6 weeks); p^2^—effect of group (group A, group B); p^3^—group × measurement effect. Statistically significant results are marked in bold.

**Table 4:** Quality of life measured pre-intervention, after 4 weeks, and after 6 weeks using SF-36.

Outcome Measurement	Measurement Time	Group A	Group B	p^1^	p^2^	p^3^
SF-36 Physical functioning	Pre-intervention	58.34	70	**0.083**	0.263	**0.061**
	After 4 weeks	61.67	91.67			
	After 6 weeks	50	88.33			
SF-36 Role limitations due to physical health	Pre-intervention	66.67	100	0.397	0.19	0.79
	After 4 weeks	50	91.67			
	After 6 weeks	41.67	91.67			
SF-36 Role limitations due to emotional health	Pre-intervention	100	88.89	0.624	0.358	0.4
	After 4 weeks	97.22	97.22			
	After 6 weeks	100	77.78			
SF-36 Energy/fatigue	Pre-intervention	46.67	65	**0.056**	0.731	**0.099**
	After 4 weeks	82.5	70.3			
	After 6 weeks	66.67	53.33			
SF-36 Emotional well-being	Pre-intervention	72	73.34	0.19	0.157	0.23
	After 4 weeks	73.34	54.67			
	After 6 weeks	84	66.67			
SF-36 Social functioning	Pre-intervention	79.1	91.67	**0.001**	**0.08**	**0.001**
	After 4 weeks	22.5	55			
	After 6 weeks	87.5	87.5			
SF-36 Pain	Pre-intervention	34.16	75	0.56	0.14	0.18
	After 4 weeks	50	69.16			
	After 6 weeks	26.67	75.84			
SF-36 General health	Pre-intervention	28.33	44.8	0.92	0.432	0.57
	After 4 weeks	21.67	45			
	After 6 weeks	23.33	48.33			
SF-36 Total	Pre-intervention	58.05	71.5	0.78	0.23	0.78
	After 4 weeks	58.1	75.8			
	After 6 weeks	56.6	73.8			

p^1^—effect of measurement (pre-intervention, after 4 weeks, after 6 weeks); p^2^—effect of group (group A, group B); p^3^—group × measurement effect. Statistically significant results are marked in bold.

## Data Availability

The data presented in this study are available on request from the corresponding author. The data are not publicly available due to confidential patient information.
